# Association of screen time and physical activity with health-related quality of life in Iranian children and adolescents

**DOI:** 10.1186/s12955-018-1071-z

**Published:** 2019-01-05

**Authors:** Nazgol Motamed-Gorji, Mostafa Qorbani, Fatemeh Nikkho, Mojgan Asadi, Mohammad Esmaeil Motlagh, Omid Safari, Tahereh Arefirad, Hamid Asayesh, Rasool Mohammadi, Morteza Mansourian, Roya Kelishadi

**Affiliations:** 1 Chronic Diseases Research Center, Endocrinology and Metabolism Population Sciences Institute, Tehran University of Medical Sciences, Tehran, Iran; 2 Non-communicable Diseases Research Center, Alborz University of Medical Sciences, Karaj, Iran; 3 Endocrinology and Metabolism Research Center, Endocrinology and Metabolism Clinical Sciences Institute, Tehran University of Medical Sciences, Tehran, Iran; 4 Osteoporosis Research Center, Endocrinology and Metabolism Clinical Sciences Institute, Tehran University of Medical Sciences, Tehran, Iran; 5 Department of Pediatrics, Ahvaz Jundishapur University of Medical Sciences, Ahvaz, Iran; 6 Evidence-Based Phytotherapy and Complementary Medicine Research Center, Alborz University of Medical Sciences, Karaj, Iran; 7 Department of Exercise Physiology, Science and Research Branch, Islamic Azad University, Tehran, Iran; 8 Department of Medical Emergencies, Qom University of Medical Sciences, Qom, Iran; 9 Department of Epidemiology, School of Public Health, Shahid Beheshti University of Medical Sciences, Tehran, Iran; 10 Department of Health Education and Promotion, School of Health, Iran University of Medical Sciences, Tehran, Iran; 11 Department of Pediatrics, Child Growth and Development Research Center, Research Institute for Primordial Prevention of Non-communicable Diseases, Isfahan University of Medical Sciences, Isfahan, Iran

**Keywords:** Health-related quality of life, Physical activity, Screen time, Children and adolescents

## Abstract

**Background:**

Health-related quality of life (HRQoL) is a multidimensional concept with significant effects and children and adolescence; while physical activity (PA) and screen time (ST) have been suggested as its probable predictors. Present study aims to investigate the association of PA, ST and their combination, with HRQoL in a nationally-representative sample of Iranian children and adolescents.

**Methods:**

As for the estimated sample size**,** 25,000 students aged 6–18 years were selected via multi-stage cluster sampling from 30 provinces of Iran. Sociodemographic data was obtained by using the questionnaire of the World Health Organization-Global school based student health survey (GSHS). Persian Pediatric Quality of Life inventory (PedsQL) and Physical Activity Questionnaire for the pediatric age group (PAQ-A) were applied for evaluating HRQoL and PA, respectively. PA scores 1–1.9 and 2–5 were respectively considered as low and high PA. The average duration of time spent on watching TV and leisure time computer use were considered as ST behaviors. ST of less than 2 h was considered low.

**Results:**

Out of 25,000 invited individuals**,** 23,043 students (mean age: 12.5) completed the study (response rate: 92.17%). In linear regression models, ST duration had significant inverse association with total QoL (β: − 0.49, *p* < 0.05). PA showed positive significant associations with HRQoL total score (β: 1.8, *p* < 0.05). Joint association of PA and ST revealed the strongest association of “high PA-low ST” category with total HRQoL (β: 2.2, *p* < 0.05); while “high PA-high ST” showed better total HRQoL score (β: 1.3) compared to “low PA-low ST” subgroup.

**Conclusion:**

Both PA and ST are significantly and independently associated with HRQoL in Iranian children and adolescents; while the adverse effect of prolonged ST could be diminished by a high PA.

## Introduction

Health-related quality of life (HRQoL) is considered as a multidimensional concept which incorporates experiences, beliefs and perceptions of physical, psychological and social aspects of health [1]. While HRQoL has been suggested as an independent predictor of mortality in adults [2], HRQoL of children and adolescents has received accumulative attention due to its comprehensive characteristics. Significance of HRQoL assessment in children and adolescents arises from its capability to identify at risk individuals, detect health inequalities [3, 4] and act as a basis for evaluating adulthood HRQoL [5]. Therefore due to the significance enumerated for HRQoL, several studies have investigated its influential correlators in a global level.

The impact of numerous factors on HRQoL of children and adolescents have been addressed previously; namely weight status [6], body image (perceived weight status) [7], smoking status [8], socioeconomic status of the family [9] and sleeping patterns [10]. Furthermore, chronic conditions (e.g. Cystic fibrosis) could affect HRQoL of adolescents as well [11].

Low physical activity (PA) and prolonged screen time (ST) are among factors that have been suggested as predictors of HRQoL in children and adolescents. They are both suggested as unhealthy behaviors which their longitudinal trends could continue into adulthood [12]. Previous studies have mentioned low PA and high ST in Iranian children and adolescents [13–16], as the mean of vigorous PA in Iranian adolescents and children was estimated to be 0.6 h/d in one study [17, 18]. Increased ST is an indicator of sedentary behaviors, and expresses situations with low energy expenditure of energy [19], namely the time spent watching television (TV), playing video games and leisure time computer work.

The association of high ST and several medical conditions has been well-documented, including obesity [20] and non-communicable diseases [21]. It has also been the center of studies investigating its effects on psychological and mental health of adolescents [22]. The association of prolonged ST and HRQoL in adolescents has been suggested previously; and while some studies have reported significant impact of ST [23], some researches reject such association [24, 25]. Similar to ST, PA has been addressed in studies focusing on mental and psychosocial health of adolescents [26, 27]. Regarding HRQoL, the majority of the literature agree on a positive effect of PA [28, 29], while few reject this association [30].

As time has passed, Iranian children/adolescents’ usage of modern technology (like TV and private computers) in their leisure time has increased, while fewer Iranian youths tend to participate in physical activities with appropriate energy consumption [31]. This phenomenon mostly results from chaotic modernization and frenetic urbanization of Iranian society; with examples including new apartment housings without yards, or lack of green spaces (such as safe parks) with suitable equipment for games and sport [32–34]. On the other hand, many Iranian parents appear to be incapable of controlling or supervising their children’s screen time due to certain cultural habits of raising a child in Iran [35].

While reviewing the literature, it was discovered that joint association of PA and ST has been mostly investigated regarding their effects on cardiometabolic risk factors. Few prior studies have evaluated their impact on HRQoL (with regards to confounders such as weight and gender), and it appears that researches comparing their independent effects (particularly in pediatric and adolescence populations) are scarce. In addition, most of the adolescence literature investigating ST and PA are conducted in western countries. ST and PA are concepts with high correlation, and considerable numbers of Iranian children/adolescents have sedentary behaviors similar to worldwide evidence [36]; however, no national-surveyed study has compared the impacts of high ST and low PA on Iranian adolescent HRQoL. Therefore, a gap of knowledge seems to be existing regarding this issue. Current study was developed to answer this question: “which one of these two factors (ST and PA) are more influential on HRQoL of Iranian children/adolescents?” or “With optimizing which one, could HRQoL be improved faster?”

Therefore, main aim was to compare mean QoL of two main groups of “low PA/high ST” with “high PA/low ST” in order to decide which one is closer to the more ideal group according to their HRQoL. Accordingly, present study aims to investigate the association of PA, ST and their possible combination with HRQoL in a sample of nationally representative of Iranian adolescents participated in CASPIAN-IV study. As a Subsidiary purpose, we aimed to investigate the effects of ST and PA on each subscale of HRQoL (school, social, physical and emotional) and compare these domains with each other to further clarify their association.

## Materials and methods

The fourth survey of **C**hildhood and **A**dolescence **S**urveillance and **P**revent**I**on of **A**dult **N**on communicable disease (CASPIAN-IV study) was conducted as a nation-wide surveillance system among a national representative sample of Iranian students. This survey was conducted in 2011–2012 in rural and urban regions of 30 provinces of Iran; and 13,486 students between ages of 6 to 18 participated in this study [37]. As a part of national survey of school students’ high-risk behavior of aforementioned surveillance system, the Assessment of Determinants of Weight Disorders Survey of the CASPIAN- IV study was conducted simultaneously in 2011–2012. Latter survey was performed with a corporative collaboration between the Ministry of Health and Medical Education, Ministry of Education and Training, Alborz University of Medical Sciences, and Child, Growth and Development Research Center affiliated with Isfahan University of Medical Sciences. Methodology and early findings of aforementioned study was described previously in a distinct study [38]. Data obtained from Weight Disorders Determinants Survey was used in the current project.

### Study population and sampling method

Total sample size for Assessment of Determinants of Weight Disorders Survey of the CASPIAN- IV study was determined according to a proportion estimation formula. In order to reach the maximum sample size, prevalence was considered 0.5, while precision was held as 0.085 and type I error as 0.05. The estimated sample size (133 subjects) was multiplied by level of education (elementary, secondary and high school), living area (urban and rural), and an attrition rate of 5%. Thus, the sample size was calculated as 830 students in each province and totally 25,000 students were invited at national level. The data from one of the provinces was not available at the end of the study, therefore analysis was done on the data from 30 provinces.

Participants were selected via multi-stage cluster sampling method, from children and adolescent students aged 6 to 18 years residing in urban and rural areas of 30 provinces of Iran. In each province, stratification was conducted based on the residence area (urban/rural) and school levels (elementary/junior-high/high school). The sampling was performed in accordance to total number of students of each province. Sex ratio was considered equal; in other words, equal numbers of male and female students were to be selected in each cluster. Furthermore, the ratios in urban and rural areas were kept proportionate according to the population of urban and rural students. Therefore the number of selected individuals in rural/urban areas and in each school grade were divided proportionally to the population of students in each grade. Sampling with equal numbers of clusters was used to make the required sample size in each province.

The list of students in each province (obtained from the information bank of the Ministry of Education) was used as the sampling frame. In each province, all of the schools were ordered by level and name of the school, and all of the students were ordered in each school level by their name (alphabetically). After determining clusters for each province, 10 students were selected serially in each cluster. In order to reach the total sample size of 25,000, 830 students were estimated from each province. Ultimately, 83 clusters of 10 subjects in each of the provinces and a total of 25,000 students were selected for the study. Therefore selected students and one of their parents were invited by telephone to participate in the study by showing up to school on certain days specifically assigned for the data gathering [38].

### Measurements and definition of terms

Data gathering of the surveillance program was performed in two steps. At first, survey team members (who were trained health-care professionals) explained the steps of the ongoing survey. Afterwards, a questionnaire was introduced to the students and their parents, which was then filled by them (Parental questionnaire only included questions regarding the nutrition of the students, a section which was not used in our study). This questionnaire was developed out of “World Health Organization Global school-based student health survey (GSHS) questionnaire”, “Physical Activity Questionnaire for Adolescents (PAQ-A)” and “Adolescent Core version of the Pediatric Quality of Life (PedsQL)”. The validity and reliability of this questionnaire was assessed and confirmed previously; Cronbach alpha of the whole questionnaire was estimated to be 0.97 and the Pearson correlation coefficient of the test–retest phase was 0.94. GSHS questionnaire included questions about socio-demographic characteristics of the family, namely parental education, owning state of a car or house, physical activity and health-related quality of life [39].

Pediatric Quality of Life inventory (PedsQL™ 4.0™ 4.0) Generic Core Scales was applied for evaluating quality of life scores in participants. Persian version of this questionnaire was proved valid in Iranian pediatric samples in prior studies [40]. Health-Related Quality of Life (HRQoL) of the students was assessed using the Adolescent Core version of the Pediatric Quality of Life (PedsQL). PedsQL is a self-reporting questionnaire comprising 23 items; and its four main scales included physical functioning (8 items), emotional functioning (5 items), social functioning (5 items) and school functioning (5 items). These 23 items were evaluated as a list, and the student was required to clarify the degree to which each item has been bothering him/her during past one month. Each statement was scored from 0 (participant has never had a problem regarding the statement during past month) to 4 (participant almost always has had a problem regarding the statement during past month). Total scale score was calculated from the mean of all 23 items, and a psychosocial score was also derived from the mean of social, emotional and school functioning scales (15 items) scores.

Evaluation of physical activity (PA) was performed using the physical activity questionnaire for adolescents (PAQ-A). PAQ-A is a modified version of the physical activity questionnaire for children (PAQ-C), which was developed for evaluation of children’s general levels of physical activity [41]. Validity and reliability of these questionnaires have also been approved in Iranian populations previously [42]. PA scores were obtained through different questions on student’s activities in last seven days. These questions encompassed different activities of participants (including different sports, activities leading to harder breathing or an increase in heart rate) during a wide range of periods (namely physical education class, school breaks, lunch times, after school, in the evenings, on weekends and in general during last week). This variable was classified as low PA level (PAQ-A score: 1–1.9), and high PA level (PAQ-A score: 2–5) [43].

In current study, ST was evaluated and obtained through two determinants via the GSHS questionnaire; time spent on watching TV (TV time), and time spent on leisure time computer working (Computer/PC time). Prolonged values of each of these variables was defined as a (TV or PC) time more than 2 h a day; subsequently total ST was cumulatively computed and categorized to high (≥ 2 h per day) and low (< 2 h per day) according to the international ST recommendations [44, 45].

In the second step, physical measurements were performed by skilled nurses under standard protocols using calibrated instruments. Weight was assessed to the nearest 200 g via a scale placed on the ground. Participants were weighed barefoot and in a light clothing condition. Height was measured in a standing position and without shoes to the nearest 0.1 cm with a non-elastic band. Body mass index (BMI) was calculated by dividing weight (kg) to height squared (cm2) [38]. The World Health Organization (WHO) standard curves were used to categorize BMI into four groups of underweight (BMI less than 5th percentile for age and gender), normal weight (BMI between 5th and 85th percentiles for age and gender), overweight (BMI between 85th and 95th percentiles for age and gender) and obese (BMI greater than the 95th percentile for age and gender).

According to analytical methods and variable selection of previous evidence in the “Progress in the International Reading Literacy Study” (PIRLS) and by using Principle Component Analysis (PCA) method, socioeconomic status (SES) score was computed based on family properties (Such as owning a private car or personal computer), parents’ job, education level of parents and student’s school type (private or governmental). Mentioned determinants were used to generate one main variable called SES score. This main variable explained 59% of total variance. Thereafter, students were classified in low, moderate and high SES based on this score.

### Statistical analysis

Continuous variables are represented with scores and standard deviation (*SD*), while categorical variables are reported with number (*n*) and percentage *(%).* Mean of age in two genders were computed using T- test. Association of categorical variables with sex was investigated using Chi-square test. Linear regression analysis was performed for evaluating the association between different QoL scores with PA, ST and their combination. Two models were defined for each association; Model I represents the unadjusted association between ST, PA and their combined subscales. Model II represents the association adjusted for age, sex, living area, BMI, SES, in addition to adjustment for physical activity (regarding ST) or screen time (regarding PA). Data of linear regressions are presented with β-coefficient.

Data was analyzed using survey data analysis methods to account effect of multistage cluster sampling in the STATA Corp. 2011 (Stata Statistical Software: Release 12. College Station, TX: Stata Corp LP. Package). *P*-value < 0.05 was considered as statistically significant. Missing data of this project constituted less than 5% of the data. Therefore it was considered at random and imputed using Amelia package program.

### Compliance with ethical standards

Current study was performed according to the declaration of Helsinki, and was approved ethically by the ethics committees and national and provincial regulatory organizations. The ethical code for this study was 5429–90. Participants were thoroughly explained about aims and protocols of the study, and were assured that their responses would remain anonymous and confidential. Participation in the study was voluntary, and participants were aware of their right to withdraw from the study at any time. Oral assent and written informed consent were obtained (prior to inclusion in the study) from students and one of their parents, respectively.

## Results

Current study included 23,043 students (50.8% boys: 11,706 boys and 11,337 girls) between 6 and 18 years old (response rate: 92.17%). Table 1 demonstrates the demographic characteristics of participants according to gender. Mean age of participants was 12.5 (±3.3), and 73.5% resided in urban areas. In general, mean BMI was 18.8 and 6.5% of the population were obese. Prevalence of obesity was significantly higher in boys compared to girls. Higher percentage of boys (86.2%) were significantly physically active compared to girls; PA score was also higher in boys with 2.2 compared to 1.8 in girls. 40.9% of total population had high screen time (ST ≥ 2 h) and the values were significantly different between girls and boys (girls>boys). Total QoL mean score was 78.3 and scores of all components of QoL (except social QoL) were significantly higher in boys compared to girls (*P*-value< 0.001).

**Table 1:** General characteristics of participants according to gender

Variables	Girls	Boys	Total	*P* value
Age (Year)^a^	12.6(3.3)	12.4(3.3)	12.5(3.3)	< 0.001
Height (cm)^a^	146.2(16.1)	148.5(19.7)	147.4(18.1)	< 0.001
Weight (Kg)^a^	41.9(15.5)	43.1(18.3)	42.5(17)	< 0.001
BMI (kg/m2)^a^	18.9(4.5)	18.7(4.4)	18.8 (4.4)	< 0.001
BMI score^a^	0.03(1.01)	− 0.03(0.98)	< 0.001(1)	< 0.001
ST(hour)^a^	1.97(1.4)	1.8(1.1)	1.9 (1.2)	< 0.001
PC time (hour)^a^	1.1 (1.4)	0.76 (1.1)	0.93(1.3)	< 0.001
TV time (hour)^a^	2.6(1.8)	2.5(1.4)	2.6(1.6)	< 0.001
PA score^a^	1.8(0.73)	2.2(0.76)	2(0.78)	< 0.001
Psychosocial QoL score^a^	74.7(17.9)	81(14.3)	77.9(16.5)	< 0.001
School QoL score^a^	71.4(17.3)	79.8(14.3)	71.7(16.4)	< 0.001
Social QoL score^a^	90(14.1)	90(14.4)	90(14.25)	0.32
Emotional QoL score^a^	75.4(20.2)	81(18.2)	78.2(19.5)	< 0.001
Physical QoL score^a^	83.9(14.9)	84.6(14.4)	84.2(14.7)	< 0.001
Total QoL score^a^	75.6(17.8)	81(13.9)	78.3(16.17)	< 0.001
PA category (n)^b^				
Active	7485(66.6)	9992(86.2)	17,477(76.5)	< 0.001
Inactive	3758(33.4)	1605(13.8)	5363(23.5)	
Region (n)^b^				
Urban	8387(75)	8271(72)	16,658(73.5)	0.17
Rural	2796(25)	3217(28)	6013(26.5)	
PC category (n)^b^				
< 2 h	6971 (75.6)	7412(83.9)	14,383(79.7)	< 0.001
> =2 h	2247(24.4)	1418(16.1)	3665(20.3)	
TV category (n)^b^				
< 2 h	3193(30.1)	3273(29.7)	6466(29.9)	0.64
> =2 h	7405(69.9)	7756(70.3)	15,161(70.1)	
ST category (n)^b^				
< 2 h	6151(57.3)	6768(60.8)	12,920(59.1)	0.0057
> =2 h	4591(42.7)	4364(39.2)	8955(40.9)	
General obesity (n)^b^				
No	10,743(94.7)	10,813(92.3)	(21556)93.5	< 0.001
Yes	594(5.3)	898(7.7)	1492(6.5)	
SES (n)^b^				
Low	3136(33.6)	3307(33.8)	6443(33.4)	0.80
Moderate	3175(33.5)	3250(33.2)	6425(33.4)	
High	3174(33.5)	3225(33)	6399(33.2)	

*BMI* body mass index, *ST* screen time, *PC* personal computer, *TV* television, *PA* physical activity, *QoL* quality of life, *SES* socioeconomic status

^a^Data are presented as mean (SD)

^b^Data are presented as number (%)

Table 2 represents scores of different components of QoL according to binary situations of ST and PA. As it can be implied from the table, all QoL scores were significantly higher in high PA with total mean score of 82.5 compared to 79.1 in low PA (*P*-value< 0.001). While most of the QoL components had significantly higher scores in low ST category, these values were insignificant for social and physical QoL components.

**Table 2:** Mean (SD) of quality of life component according to the level of screen time and physical activity

Variable		Psychosocial QoL score	School QoL score	Social QoL score	Physical QoL score	Emotional QoL score	Total score
Watching TV^a^	high	81.4(13.9)	78.8(14.3)	90(14.3)	84.3(14.7)	78.3(19.6)	82(13.3)
	low	81.3(14.3)	78.7(14.8)	90.2(14.2)	84(14.8)	78.2(19.3)	81.7(13.8)
	*P* value	0.74	0.68	0.16	0.9	0.54	0.91
Computer Time^a^	high	79.5 (15.1)	76.1(15.6)	89.84(14.4)	84.6(14.8)	77.4(19.7)	80.5(14.7)
	low	81.9(13.4)	79.4(13.9)	90(14.2)	84.2(14.7)	78.4(19.3)	82.4(12.9)
	*P* value	< 0.001*	< 0.001*	0.25	0.88	0.01*	< 0.001*
ST duration^a^	high	80.6(14.5)	78(14.8)	89.9(14.4)	84.2(14.9)	77.7(19.9)	81.2(14)
	low	81.9(13.7)	79.3(14.2)	90.1(14.1)	84.3(14.6)	78.6(19.2)	82.3 (13.1)
	*P* value	< 0.001*	< 0.001*	0.1	0.27	< 0.001*	< 0.001*
PA^a^	Active	82(13.8)	79.4(14.4)	90.3(13.9)	85.1(14.1)	79.5(18.7)	82.5(13.2)
	inactive	78.8(15)	76.2(15.1)	89.1(15.2)	81.4 (16.1)	74.2 (21.3)	79.1(14.5)
	*P*-value	< 0.001*	< 0.001*	< 0.001*	< 0.001*	< 0.001*	< 0.001*

*ST* screen time, *PC* personal computer, *TV* television, *PA* physical activity, *QoL* quality of life

^a^Data are presented as mean (SD)

^*^Statistically significant

Table 3 shows the association of ST with components of QoL in linear regression models. As it can be seen, ST duration demonstrated significant reverse association with total QoL and its components except for social and physical QoL. Mentioned association was significant in both continuous and binary forms of ST, and remained statistically significant after adjustment for confounding variables (age, sex, living area, BMI, PA and SES). The association of ST was strongest with psychosocial and school components of QoL in adjusted continuous model. Using linear regression models, Table 4 illustrates the association between PA and components of QoL. PA (in both continuous and dichotomous models) demonstrated positive significant associations with all components of QoL and QoL total; all of the associations remained significant after adjustment for mentioned confounding variables. The association of PA was strongest with emotional and physical components of QoL in adjusted continuous model.

**Table 3:** Association of screen time with quality of life in children and adolescents

Variable	Psychosocial QoL	School QoL	Social QoL	Physical QoL	Emotional QoL	Total QoL
Watching TV(hour)						
Unadjusted model	**−0.22 (− 0.38,-0.05)** ^**a**^	**− 0.26 (− 0.42,-0.97)** ^**a**^	**− 0.17 (− 0.33,-0.01)** ^a^	**−0.22 (− 0.38,-0.05)** ^a^	**−0.38 (− 0.6,-0.16)** ^a^	**−0.18 (− 0.34,-0.3)** ^a^
Adjusted model^b^	**− 0.19 (− 0.37,-0.02)** ^**a**^	**−0.21 (− 0.38,-0.03)** ^**a**^	**−0.21 (− 0.38,-0.04)** ^**a**^	**−0.25 (− 0.42,-0.08)** ^**a**^	**−0.33 (− 0.56,-0.1)** ^**a**^	**−0.18 (− 0.34,-0.01)** ^**a**^
Watching TV (high vs low)						
Unadjusted model	0.14 (−0.39,0.68)	0.11 (−0.44,0.67)	− 0.22 (− 0.75,0.31)	0.31 (− 0.23,0.86)	0.037 (− 0.71,0.79)	0.29 (− 0.22,0.81)
Adjusted model ^b^	0.024 (− 0.54,0.59)	0.05 (− 0.5,0.61)	−0.3 (− 0.86,0.25)	0.17 (− 0.4,0.76)	−0.15 (− 0.93,0.62)	0.17 (− 0.38,0.72)
Computer time (hour)						
Unadjusted model	**−0.86 (− 1.18,-0.53)** ^**a**^	**− 1.18 (− 1.51,-0.86)** ^**a**^	−0.01 (− 0.22,0,19)	0.23 (− 0.08,0.46)	−0.22 (− 0.53,0.08)	**−0.68 (− 1.0,-0.36)** ^**a**^
Adjusted model ^b^	**− 0.67 (− 0.99,-0.35)** ^**a**^	**−0.85 (− 1.16,-0.53)** ^**a**^	−0.09 (− 0.31,0.138)	0.14 (− 0.09,0.38)	−0.09 (− 0.4,0.23)	**−0.55 (− 0.87,-0.23)** ^**a**^
Computer time (high vs low)						
Unadjusted model	**−2.39(− 3.3,-1.47)** ^**a**^	**− 3.22 (− 4.13,-2.31)** ^**a**^	− 0.19 (− 0.94,0.54)	0.36 (− 0.39,1.1)	**− 0.95 (− 1.89,-0.01)** ^**a**^	**−1.9 (− 2.82,-0.99)** ^**a**^
Adjusted model ^b^	**− 1.81 (−2.72,-0.91)** ^**a**^	**− 2.24 (− 3.1,-1.35)** ^**a**^	− 0.38 (− 1.13,0.36)	0.03 (− 0.73,0.81)	−0.59 (− 1.59,0.4)	**− 1.5 (− 2.4,-0.63)** ^**a**^
ST duration (hour)						
Unadjusted model	− 0.64 (− 0.88,-0.4)	−0.77 (− 1.02,-0.53)	−0.16 (− 0.38,0.059)	−0.15 (− 0.37,0.06)	−0.5 (− 0.81,-0.19)	−0.55 (− 0.78,-0.32)
Adjusted model ^b^	− 0.55 (− 0.79,-0.3)	−0.6 (− 0.83,-0.36)	−0.23 (− 0.47,0.008)	−0.21 (− 0.44,0.007)	−0.44 (− 0.75,-0.12)	−0.49 (− 0.72,-0.25)
ST duration (high vs low)						
Unadjusted model	**−1.23 (− 1.79,-0.68)** ^**a**^	**− 1.3 (− 1.87,-0.73)** ^**a**^	−0.27 (− 0.78,0.24)	−0.13 (− 0.66,0.4)	**−0.95 (− 1.66,-0.24)** ^**a**^	**−1.0 (− 1.5,-0.5)** ^**a**^
Adjusted model ^b^	**− 1.15 (− 1.7,-0.59)** ^**a**^	**−1.07 (− 1.63,-0.52)** ^**a**^	−0.42 (− 0.95,0.1)	−0.35 (− 0.91,0.19)	**−0.96 (− 1.68,-0.23)** ^**a**^	**−1 (− 1.5,-0.48)** ^**a**^

*ST* screen time, *PC* personal computer, *TV* television, *QoL* quality of life

Data are presented as β coefficient (95% CI)

^a^Statistically significant

^b^Adjusted for age, sex, living area, body mass index, socioeconomic status and physical activity

**Table 4:** Association of physical activity with quality of life in children and adolescents

Variable		Psychosocial QOL	School QoL	Social QoL	Physical QoL	Emotional QOL	Total QoL
PA score	Unadjusted model	2.2(1.8,2.6)^a^	2.3(1.89,2.7)^a^	0.8(0.51,1.1)^a^	2.2(1.9,2.6)^a^	3.3(2.8,3.8)^a^	2.3(1.9,2.6)^a^
	Adjusted model ^b^	1.5(1.12,1.89)^a^	1.0(0.65,1.4)^a^	1.05 (0.68,1.4)^a^	2.4(2.0,2.8)^a^	2.6(2.1,3.1)^a^	1.8(1.4,2.2)^a^
PA(active/inactive)	Unadjusted model	3.24(2.5,3.9)^a^	3.1(2.4,3.9)^a^	1.2(0.56,1.79)^a^	3.7(3,4.36)^a^	5.4(4.4,6.3)^a^	3.4(2.6,4.1)^a^
	Adjusted model ^b^	1.9(1.1,2.5)^a^	0.97(0.28,1.7)^a^	1.3(0.63,1.9)^a^	3.7(2.9,4.4)^a^	3.9(2.9,4.9)^a^	2.3(1.6,3.1)^a^

*QoL* quality of life, *PA* physical activity

Data are presented as β coefficient (95% CI)

^a^Statistically significant

^b^Adjusted for age, sex, living area, body mass index, socioeconomic status and screen time

Adjusted joint association of ST and PA with QoL components is displayed in Table 5, using a combined (joint) variable of ST-PA. Comparing different modes of the joint variable by their total QoL suggests the strongest association of HRQoL with “high PA-low ST” category (β coefficient = 2.2). Among QoL components in mentioned subcategory, emotional and physical QoL have the strongest associations. Participants with joint combination of “high PA-high ST” display a better total QoL (with β-coefficient of 1.3) compared to “low PA-low ST” subgroup (which was considered as reference in this model analysis). As the subgroup with worst total QoL, “low PA-high ST” had significant inverse association with emotional and physical QoL components.

**Table 5:** Joint association of physical activity and screen time with quality of life in children and adolescents

Variable		Psychosocial QOL	School QoL	Social QoL	Physical QoL	Emotional QOL	Total QoL
Joint association of PA-ST in adjusted model^b^	Low PA-Low ST	Reference	Reference	Reference	Reference	Reference	Reference
	Low PA-High ST	−1.1 (−2.2, 0.1)	−0.72 (− 1.9, 0.4)	− 0.79 (− 1.9, 0.31)	**−1.8 (−3.0,-0.7)+**	**− 1.8 (− 3.4,-0.21)** ^**a**^	**− 1.3 (− 2.4,-0.21)** ^**a**^
	High PA-Low ST	**1.9 (1.0, 2.7)** ^a^	1.1 (0.3, 2.0)^a^	1.1 (0.3, 1.9)^a^	**2.9 (2.0,3.8)** ^**a**^	**3.5 (2.3,4.7)** ^**a**^	**2.2 (1.3,3.1)** ^**a**^
	High PA- High ST	**0.7 (− 0.2, 1.7)** ^a^	0.0 (− 0.9,0.9)	0.84 (− 0.01, 1.7)	**3.1 (2.1,4.1)** ^**a**^	**2.8 (1.5,4.1)** ^**a**^	**1.3 (0.4,2.3)** ^**a**^

*QoL* quality of life, *PA* physical activity, *ST* screen time;

Data are presented as β coefficient (95% CI)

^a^Statistically significant

^b^Adjusted for age, sex, living area, body mass index, socioeconomic status

## Discussion

Present study investigated the association of PA and ST with different subclasses of HRQoL in a representative sample of Iranian children and adolescents. Our findings suggest that both PA and ST are significantly associated with overall HRQoL scores. In other words, children and adolescents with higher amounts of PA are more likely to have better HRQoL scores; and individuals with higher ST hours (weather as TV or PC time) were more frequently reporting poorer HRQoL. The association between ST and PA with HRQoL domains was observed to be independent of other confounding factors. Furthermore, the effects of ST and PA on HRQoL were indicated to be independent of each other as well, as their influence was omitted from the association with HRQoL. Therefore, both ST and PA are independently and significantly associated with HRQoL in adolescents. Gender was also controlled in association among ST, PA and HRQoL, while the association preserved to be significant after controlling for its effect. However, in general boys tended to have significantly higher HRQoL scores, while reporting higher PA scores and lower ST.

Our findings on the independent association of both ST and PA are in agreement with several prior studies investigating HRQoL in adolescents [46, 47]. HRQoL was evaluated by different measures in mentioned studies. Similar to current study, three adolescent researches investigated the role of ST and PA with HRQoL using PedsQL measurement. In a cross-sectional 2012 study by Lacy et al., both high PA and low ST were associated with higher HRQoL scores; and these associations were independent of participants’ weight [23]. Another 2012 study conducted by Gopinath et al. as a cohort followed adolescents during 5 years, and demonstrated that both PA (positively) and ST (inversely) affect adolescents HRQoL [28]. These findings were in agreement with their following 2014 study, in which (using general linear mode) it was suggested adolescents with more life style risk factors (including low PA and high ST) are at greater risk for experiencing poorer HRQoL [48]. Several studies have suggested a role for gender direction in the association of PA/ST and HRQoL. Other Iranian studies have also reported lower PA scores in girls [18]. A similar study conducted in Tehran (capital of Iran) presented higher PA and HRQoL scores in boys compared to girls; but it also suggested higher ST values in them as well, which is in contrast with our findings [49].

By contrast, various studies suggested that no association exists between ST and PA with HRQoL. Borras et al. investigated the roles of both ST and PA in adolescence HRQoL using CHIP-CE/PRF measurement. While ST was considered a significant predictor of HRQoL, PA showed no association with it. It was suggested that positive influence of PA observed in other studies might be justified by mediating effects of cardiorespiratory fitness, which as a result could affect HRQoL [50]. In 2010 Boyle et al. investigated the effect of PA and adolescence HRQoL using two measurements of PedsQL and EQ-5D utility score. PedsQL scores were not associated with PA levels in this study. Therefore authors suggested the positive associations observed in other studies to be mostly due to mediating roles of other confounding factors, including obesity [30]. However, Goldfield et al. showed that controlling for obesity and PA could reveal the association between ST and PedsQL scores [51]. A 2015 study investigating the joint association of ST and PA in HRQoL of adults remarked no independent impact for sedentary behaviors (a finding opposing our findings), while PA was greatly associated with HRQoL [24]. Recently Wafa et al. reported the association of PA with HRQoL in 11–13 years old Malaysian adolescents, which was disappeared after adjusting the association for BMI and gender. This study explained the distinction between contradicting findings of previous studies through cultural disparities [52]. Some studies believe that not all amounts of ST would inevitably lead to poorer life quality. They suggested that instead of a direct inverse, ST and health indicators have rather a curvilinear or U-shaped association; in which the moderate amount of ST has the best mental health or academic outcomes [25, 53].

Findings of current study indicate that ST is most strongly associated with school functioning and psychosocial functioning of adolescents. PA on the other hand, demonstrated the strongest association with emotional and physical subclasses of adolescence HRQoL. Many researches investigating the field of adolescents’ mental health are in agreement with these findings. Low PA has been suggested to correlate with anxiety and depression; mental health conditions that are mostly reflected by emotional functioning [54, 55]. Social isolation and depression are addressed as correlated features of excessive internet usage in research literature [56]. Physical functioning advancement associated with higher PA also concurs acceptably with prior studies [29, 57]. High magnitudes of ST are believed to interfere with many school aspects including sleep patterns [58]and physiological arousal [59]. A justification for ST and school functioning association is that subjects exposed to more rapid changing images are less capable of attending the slow real life, namely school events [60]. In a summary, these findings conclude that in order to improve psychosocial HRQoL in children and adolescents, the best management would be to control and reduce ST (rather than increasing their PA); while in dealing with poor physical and emotional HRQoL in children and adolescents, encouraging to increase PA would be more beneficial than decreasing ST.

In context of HRQoL domains, findings of PedsQL studies seem to be contradictory. Lacy et al. approved the strong association between PA and emotional domain of HRQoL [23]; while results of Shoup et al. confirmed the powerful association between PA and physical function domain [61]. Gopinth et al. reported the strongest association of PA with physical and social domains of HRQoL, while emotional and psychosocial functions showed no significant association [28]. This lack of association had been supported by some previous adult studies [62, 63], and contradicts the results of current study. ST on the other hand, was significantly associated with all domains of PedsQL in Gopinath study. While our results failed to prove a significant association between social domain of PedsQL and ST, Gopinath et al. suggested an inverse effect of ST as a time devoid of family and peer interactions which could result in poorer HRQoL [28].

One interesting finding of current study concerns joint effects of ST and PA. Combined (joint) variable ST-PA was designed for the purpose of comparing the intensity of effects of ST and PA on HRQoL in adolescents. “High PA-high ST” situation was associated with better total HRQoL, compared to “low PA-low ST” condition. In other words, higher quantities of PA is capable of overshadowing the negative effect of high ST on HRQoL and result in a better HRQoL. Therefore it is suggested that the effect of PA on HRQoL could precede the effects of ST. Literature review reveals scarce evidence in the context of ST or PA’s advantage in affecting HRQoL. In 2009 Hamer et al. investigated the joint effects of ST and PA on HRQoL in a sample of UK adolescents. Results of this study indicated the independent effects of each variable on SDQ-HRQoL scores of adolescents. Results of mentioned research expressed a more favorable HRQoL in “high ST-high PA” combination compared to “low ST-low PA”; and therefore it is consistent with our findings [64]. Dalton et al. investigated the association in American adolescents. Similar to present study, HRQoL of adolescents was assessed via PedsQL scoring. Authors reported significant independent effects for both ST and PA, and suggested the influence of ST to dominate PA effect; which contradicts current study [65]. The reason for this conflict could be justified by addressing the difference in studies’ analyses. Aforementioned study did not use combined effects to compare the impacts of ST and PA, and prioritized ST as a consequence of its larger β-coefficient. It should be noted that since ST and PA are not of same modality, their unit values should not be compared to each other. Another study also investigated the joint association of ST and PA with HRQoL of Australian adults. Similar to current findings, combined category of ST and PA demonstrated the greater impact of PA in influencing the HRQoL scores [66].

To the best of our knowledge, present study is the first Iranian research that investigated the joint association of PA and ST on PedsQL-HRQoL in a large population-based pediatric group. It is also the first study of its kind in the Middle East and North Africa (MENA) region. Several studies have addressed the impacts of ST and PA on HRQoL in children and adolescents; but mostly indicated their effects without comparing them together. Moreover, studies investigating HRQoL via PedsQL scoring seem to be scarce. Therefore, further studies (particularly in Middle-Eastern children and adolescents) are required to clearly subject this significant topic.

## Strengths and limitations

The main limitation of current study is the cross-sectional design, which makes it difficult for causal inference among PA, ST and HRQoL; therefore these associations might be bidirectional. Another limitation is the subjective method of assessing ST and PA, which could be considered imprecise compared to objective methods. Studying the effect of sleeping time could have been very important in the context of QoL, since ST is often negatively associated with sleep time. Therefore not assessing it is one this study’s limitations.

On the other hand, the main strengths of this study are its large sample size and nationwide coverage. Measurement of PA, ST and HRQoL by well-validated questionnaires such as PAQ-A, GSHS and PedsQL in aforementioned large population could underscore subjective assessment of these variables. This study is novel due to the fact that it addresses the effects of PA and ST across all domains of HRQoL, which were scarcely investigated in previous literature.

## Conclusion

Current study revealed that both PA and ST can significantly predict HRQoL in Iranian adolescents. These associations were independent of other factors including gender, weight status, socioeconomic status and the effect of either ST or PA on each other. Therefore, these findings suggest PA and ST as independent HRQoL determinants. While ST was mostly associated with school and psychosocial functioning, PA showed the strongest association with emotional and physical functioning subgroups of PedsQL-HRQoL. Joint association of ST and PA revealed the highest HRQoL scores in “high PA-low ST” category. Although improving both PA and ST in adolescents would definitely be considered advantageous, it appears that effects of high ST could be dominated by optimal PA. Therefore, improving PA rather than ST would be more beneficial to children and adolescents regarding a more optimal HRQoL. In a national planning level and in terms of HRQoL, designing behavioral amendment protocols in children and adolescents entails great attention to PA, more than it requires amending ST and sedentary behaviors. Further future studies on PA and ST of adolescents (especially in MENA region) are of utmost significance due to the deficiency of knowledge on this matter.

## Abbreviation

GSHS: Global school-based student health survey
GSHS: World Health Organization-Global school based student health survey
HRQoL: Health-related quality of life
PA: Physical activity
PAQ-A: Physical Activity Questionnaire for the pediatric age group
PedsQL: Persian Pediatric Quality of Life inventory
ST: Screen time
